# Activation of Protease-Activated Receptor 2 Induces VEGF Independently of HIF-1

**DOI:** 10.1371/journal.pone.0046087

**Published:** 2012-09-25

**Authors:** Jeppe Grøndahl Rasmussen, Simone Elkjær Riis, Ole Frøbert, Sufang Yang, Jens Kastrup, Vladimir Zachar, Ulf Simonsen, Trine Fink

**Affiliations:** 1 Department of Biomedicine, Pulmonary and Cardiovascular Pharmacology, Aarhus University, Aarhus, Denmark; 2 Laboratory for Stem Cell Research, Aalborg University, Aalborg, Denmark; 3 Department of Cardiology, Örebro University Hospital, Örebro, Sweden; 4 Animal Reproduction Institute, Guangxi University, Nanning, China; 5 Cardiac Stem Cell Laboratory, The Heart Centre, Rigshospitalet, Copenhagen University Hospital, Copenhagen, Denmark; Virginia Commonwealth University Medical center, United States of America

## Abstract

**Background:**

Human adipose stem cells (hASCs) can promote angiogenesis through secretion of proangiogenic factors such as vascular endothelial growth factor (VEGF). In other cell types, it has been shown that induction of VEGF is mediated by both protease activated receptor 2 (PAR2) and hypoxia inducible factor 1(HIF-1). The present study hypothesized that PAR2 stimulation through activation of kinase signaling cascades lead to induction of HIF-1 and secretion of VEGF.

**Methodology/Principal Findings:**

Immunohistochemistry revealed the expression of PAR2 receptors on the surface of hASCs. Blocking the PAR2 receptors with a specific antibody prior to trypsin treatment showed these receptors are involved in trypsin-evoked increase in VEGF secretion from hASCs. Blocking with specific kinase inhibitors suggested that that activation of MEK/ERK and PI3-kinase/Akt pathways are involved in trypsin-eveoked induction of VEGF. The effect of the trypsin treatment on the transcription of VEGF peaked at 6 hours after the treatment and was comparable to the activation observed after keeping hASCs for 24 hours at 1% oxygen. In contrast to hypoxia, trypsin alone failed to induce HIF-1 measured with ELISA, while the combination of trypsin and hypoxia had an additive effect on both VEGF transcription and secretion, results which were confirmed by Western blot.

**Conclusion:**

In hASCs trypsin and hypoxia induce VEGF expression through separate pathways.

## Introduction

The transplantation of human adipose-derived stem cells (hASCs) to induce angiogenesis is increasingly recognised as a therapeutic modality in the treatment of ischemic disease [1], [2], [3]. In a previous study, we found that both hypoxic culture as well as treatment with trypsin increases the pro-angiogenic potential of hASCs [4]. The angiogenic effect induced by hASCs is mainly paracrine, exerted through cytokines, such as the vascular endothelial growth factor (VEGF) [5]. Hence, there is great interest in attempting to increase VEGF expression in order to optimise the effect of transplanted mesenchymal stem cells [6]–[8].

VEGF has been shown to be induced both by activation of protease activated receptor 2 (PAR2) signalling and by the transcription factor hypoxia inducible factor 1 (HIF-1) [9], [10], [11]. PAR2 is a G-protein coupled receptor that is activated by proteolytic cleavage of a tethered ligand, and is known to be activated by trypsin [12], [13], [14]. Previous studies have found that different kinase cascades are implicated in PAR2 signaling [9], [15], [16]. Thus, PAR2 was found to activate both the PI3K/Akt and MEK/ERK pathways in GI epithelial cells [17], mainly the Rho/ROCK pathway in lung epithelial cells [18], and only the MEK/ERK pathway in glioblastoma cells [19]. PAR2 is not expressed in all tissues [20], and so far it is unclear whether PAR2 are expressed in mesenchymal stem cells.

In contrast, HIF-1 has so far been found in most cell types and tissues. HIF-1 is a master regulator in oxygen homeostasis and drives the expression of a plethora of genes involved in metabolism and angiogenesis, including VEGF. HIF-1 is a heterodimer comprised of the subunits HIF-1α and the aryl hydrocarbon receptor nuclear translocator (ARNT). In normoxic conditions HIF-1α is continuously degraded. In hypoxia, however, HIF-1α is stabilized, and dimerizes with ARNT to form HIF-1 [21]. Interestingly, is has recently been shown, that even in normoxia activation of PI3K and ERK pathways may stabilize HIF-1α thus leading to induction of VEGF [22], [23]. Moreover, that hypoxia and PAR2 activation may act synergistically in the promotion of angiogenesis and that there could be possible crosstalk between the protease-activated and the hypoxia-activated pathways [24], [25]. Therefore, we hypothesized that PAR2 stimulation through activation of kinase signaling cascades may lead to induction of HIF-1 and secretion of VEGF. To address the hypothesis we examined in hASCs the expression and the effect of stimulating and blocking PAR2 receptors on VEGF, inhibitors of Rho kinase (ROCK), PI3K, and MEK were applied and phosphorylation of the downstream kinases and VEGF induction was examined. Finally, the interaction of PAR2 activation and hypoxia on VEGF and HIF-1 activation was investigated.

**Table 1:** Primer sequences for primers used in quantitative real-time RT-PCR analysis.

Gene	Forward Primer Sequence	Reverse Primer Sequence
PPIA	5′ TCC TGG CAT CTT GTC CAT G 3′	5′ CCA TCC AAC CAC TCA GTC TTG 3′
YWHAZ	5′ ACT TTT GGT ACA TTG TGG CTT CAA 3′	5′ CCG CCA GGA CAA ACC AGT AT 3′
VEGF	5′ CGA TTC AAG TGG GGA ATG G 3′	5′ CAT TGA TCC GGG TTT TAT CC 3′
PAR2	5′ TCC TCA CTG GAA AAC TGA CC 3′	5′ GGA AAA GAA AGA CCC ACA GG 3′

## Methods

### Donors

This study conforms to the Declaration of Helsinki. All patients gave written informed consent and the clinical protocol was approved by the regional Committee on Biomedical Research Ethics of Northern Jutland, Denmark (project no. VN 2005/54). The adipose tissue was obtained during elective liposuction from three healthy patients without cardiovascular disease and not receiving any medication. The patients were one male and two females aged 42, 58, and 52 years, respectively. The hASC cell lines (ASC12, 21, and 23) were established as described previously, [26] and have been characterized extensively [27], [28].

**Figure 1:**
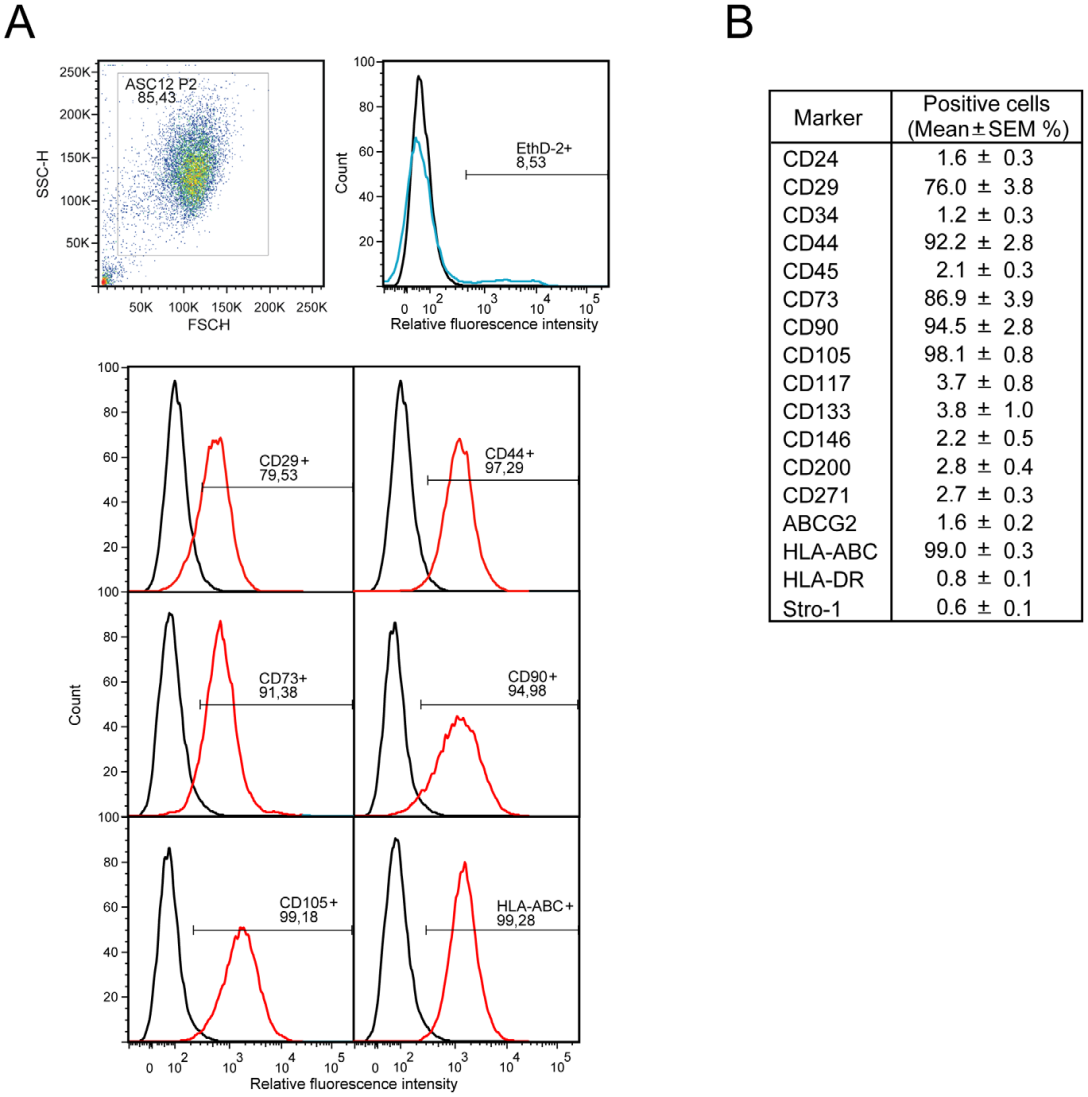
Immunophenotypical analysis of hASC lines at passage 2. (**A**) Representative distributions of positive markers expressed on the ASC12 cells are presented. (**B**) Surface markers profile was obtained as an average from ASC12, 21, and 23 lines.

### Cell Culture

The hASCs were cultured in α-MEM medium with Glutamax (Invitrogen, Taastrup, Denmark), 10% fetal bovine serum (FBS) (Europa Bioproducts Ltd., Cambridge, United Kingdom), 100 IU/ml penicillin, 100 µg/ml streptomycin, and 50 µg/ml gentamicin (all from Invitrogen). All experiments were performed with cells from all three patients using cells at passages two to five.

### FACS Analysis

Subconfluent cultures of hASCs at P2 were harvested and strained through a 70 µm filter before they were resuspended in 2% FBS and 0.1% sodium azide in PBS. The incubation with a particular antibody-fluorophore complex took place at 4°C for 30 min, after which the cells were fixed in 1% formaldehyde. Antibodies were pre-labeled with Alexa 488, R-Phycoerythrin, and Alexa 647 fluorophores using the Zenon labeling technology (Invitrogen). Primary antibodies were directed against CD24, CD29, CD73, CD117, CD133, CD146, CD200, CD271, HLA-DR (all from Abcam, Cambridge, UK), CD34, CD44, CD45, CD105, HLA-ABC (all from Dako, Glostrup, Denmark), CD90, ABCG2 (both from Santa Cruz Biotechnology, Heidelberg, Germany), and Stro-1 (Millipore Chemicon, Copenhagen, Denmark). Validity of analysis was supported by testing for viability using a LIVE/DEAD Reduced Biohazard Viability/Cytotoxicity Kit (Invitrogen). At least 10^4^ events were recorded per sample on a FACSCanto flow cytometer (BD Bioscience, Brøndby, Denmark). Data were analysed using FlowJo 7.2 software package (TreeStar, Ashland, OR).

**Figure 2:**
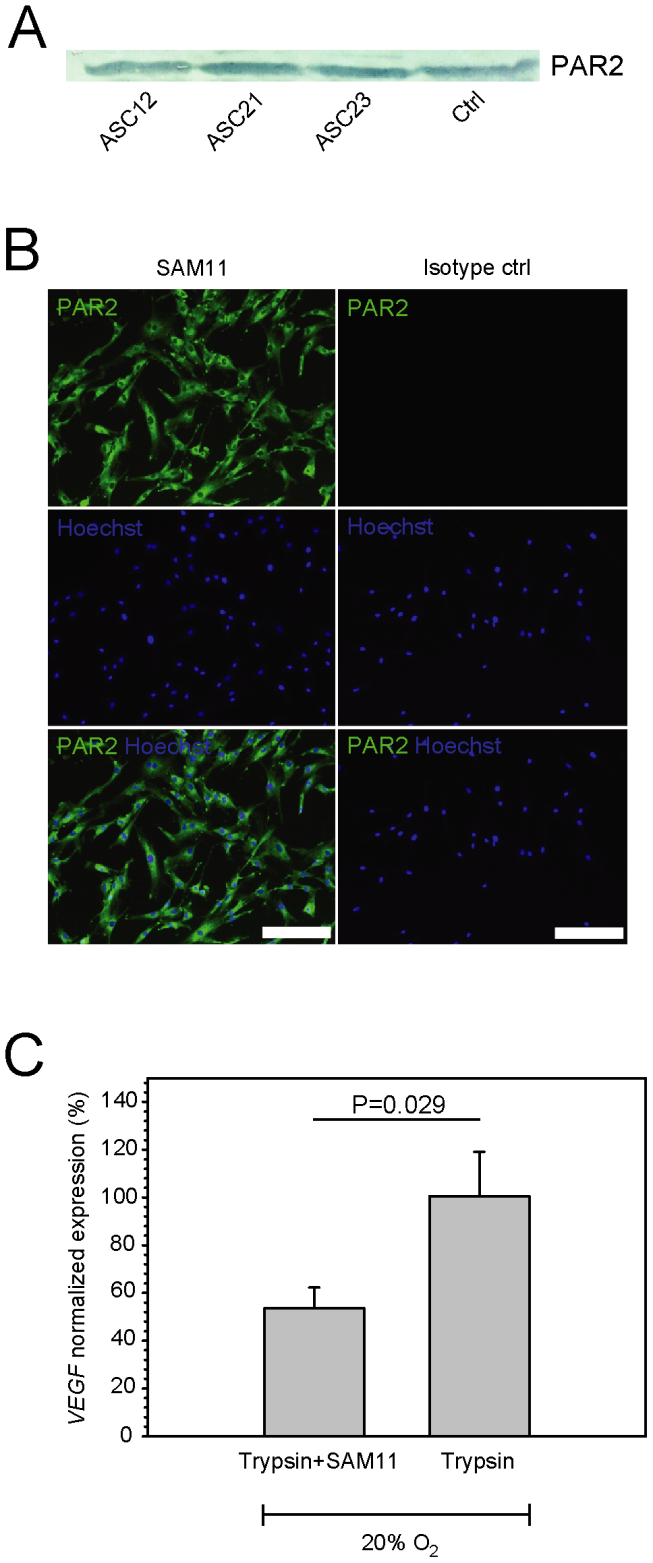
Expression of PAR2 in hASCs and its association with transcriptional activation of *VEGF*. (**A**) Detection of PAR2 in hASC lines by immunoblotting. (**B**) Expression of PAR2 by indirect immunofluorescence using SAM11 antibody. Representative pattern as detected on the surface of ASC12 cells is presented. (**C**) SAM11 antibody blocked trypsin-induced PAR2 activation, measured using real-time RT-PCR to determine *VEGF* expression levels 12 hours after trypsin exposure (n = 15). Expression levels were corrected for basal *VEGF* activity in hASCs cultured at 20% oxygen and normalised to the levels induced by trypsin. Values are represented as the mean and SEM. Scale bar indicates 200 µm. Abbreviations: PAR2, protease-activated receptor 2; VEGF, vascular endothelial growth factor; Ctrl, control (NIH 3T3 cells).

### Immunofluorescence Assay

For detection of PAR2, hASCs were seeded at 3×10^3^ per cm^2^ in 8-well glass CultureSlide (Falcon; BD Bioscience) and maintained in 20% oxygen for three days before they were fixed in 4% formaldehyde for 30 min. The cells were then reacted with 100-fold diluted mouse monoclonal antibody specific for PAR2 (SAM11; Santa Cruz Biotechnology) or matching isotype control (Dako) at 4°C overnight. The epitope sites were visualised with Alexa 488 conjugated goat anti-mouse IgG and the nuclei were counterstained with 1∶1,000 Hoechst 33342 (both from Invitogen) at 37°C for two hours. Microscopy was done using an Axio Observer Z1 (Zeiss, Göttingen, Germany) and image acquisition and processing with the aid of AxioVision 4.7 software (Zeiss).

### Treatment with Varying Concentrations of Trypsin

The cultures were passaged using a standard trypsinisation procedure based on a mixture of 0.125% trypsin and 0.01% EDTA for 5 min at 37°C. Based on manufactureŕs information regarding trypsin activity (3,600 BAEE U/mg), the 0.125% trypsin was estimated to have a concentration of 9,000 nM, using a 1 U/ml∼2 nM conversion. For the studies on effect of trypsin concentration, the hASCs were seeded in six-well plates (Costar) at 3×10^3^ cells per cm^2^, cultured for three days, and subsequently treated by either 9 nM, 90 nM, or 9,000 nM trypsin for 4 min, after which the trypsin was neutralized through addition of fresh media. Twelve hours after the trypsin treatment mRNA was harvested for transcriptional analysis. For all other experiments 9,000 nM of trypsin was used.

**Figure 3:**
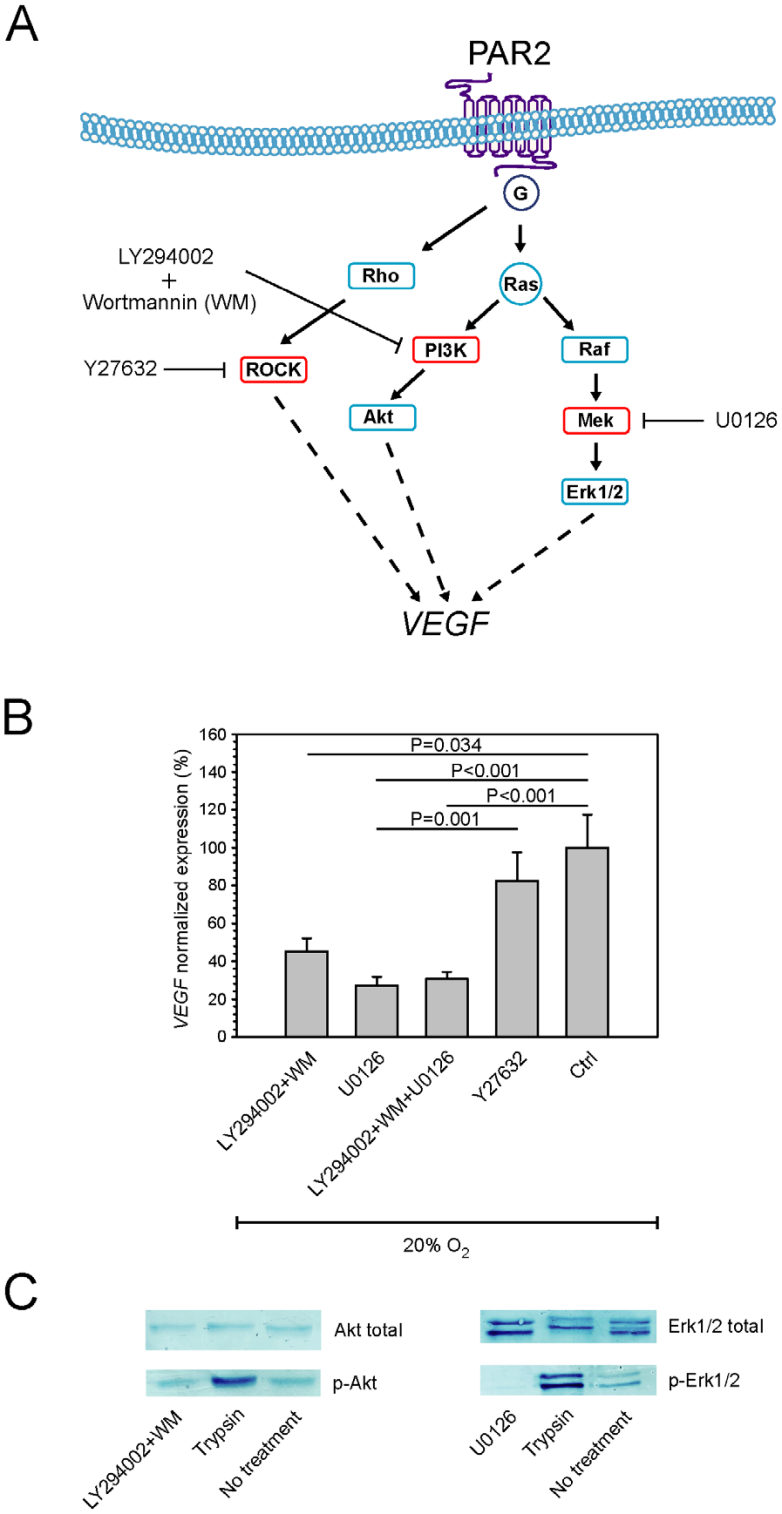
Trypsin-activated PAR2 intracellular signaling in hASCs. (**A**) Schematic rendition of signal-transduction pathways linking PAR2 and *VEGF*. (**B**) The effect of specific kinase inhibitors on suppressing trypsin-induced *VEGF* activation after 5 min trypsin exposure was assessed by real-time RT-PCR (n = 6). Expression levels were normalized to the levels induced by trypsin (Ctrl). (**C**) The effect of PI3K and Mek inhibitors on phosphorylation of Akt and Erk1/2, respectively, as a result of 5 min trypsin exposure was determined by immunoblotting. PI3K and Mek inhibitors were added 2 hours prior to trypsin exposure. Cells after a 4-day culture at 20% oxygen were used as controls (Ctrl). Representative data obtained from ASC12 cells are presented. Values are represented as the mean and SEM. Abbreviations: PAR2, protease-activated receptor 2; VEGF, vascular endothelial growth factor; Ctrl, control.

### Varying Length of Incubation after Trypsin Treatment

To determine the optimal length of incubation after trypsin treatment, the hASCs were seeded in six-well plates (Costar) at 8×10^3^ cells per cm^2^, cultured for three days, and subsequently treated by trypsin for 4 min at 37°C, after which the trypsin was neutralized through addition of fresh media. Two, six, and 12 hours after the trypsin treatment, mRNA was harvested for transcriptional analysis. For all other experiments, cells were incubated for six hours after trypsin treatment unless otherwise stated.

### Treatment by Trypsin and Hypoxia

Normoxic cultures were performed in a standard incubator in an atmosphere containing 5% CO_2_ and 20% O_2_. Hypoxic culture experiments were performed in a hypoxic workstation (Xvivo; BioSpherix, Redfield, NY) at 37°C in an atmosphere containing 5% CO_2_ balanced with nitrogen to reach oxygen concentration of 1%. The workstation allowed incubation and manipulation of the cells at continuous hypoxic conditions.

For the studies on VEGF induction, the hASCs were seeded in six-well plates (Costar) at 8×10^3^ cells per cm^2^, cultured for three days, and subsequently treated by either trypsin, hypoxia or a combination of both. For the trypsin-only and treatment, cells were treated with trypsin for 4 min at 37°C, after which the trypsin was neutralized through addition of fresh media. Six hours after the trypsin treatment, either mRNA was harvested for transcriptional analysis or the media was changed and the cells incubated an additional 12 hours, after which the media was collected for analysis of secretion of VEGF. For the hypoxic treatment, cells were transferred to 1% oxygen for 24 hours after which either mRNA was harvested or the media was changed and the cells incubated an additional 12 hours at 1% oxygen, for the analysis of the VEGF secretion. For the trypsin-hypoxia combination, the cells were cultured for 18 hours in hypoxia, treated with trypsin and refed with neutralizing media as described above, all taking place under hypoxic conditions. After 6 hours the cells were either harvested for transcriptional analysis or incubated in fresh media for 12 hours under hypoxia for analysis of VEGF secretion.

For studies on HIF-1α stabilisation by trypsin and hypoxia, the cells were seeded in T75 cell culture flasks (Costar) at a density of 8×10^3^ cells per cm^2^ and otherwise treated as described above. In addition, samples were harvested for analysis of trypsin effect 4 and 12 hours after the treatment.

**Figure 4:**
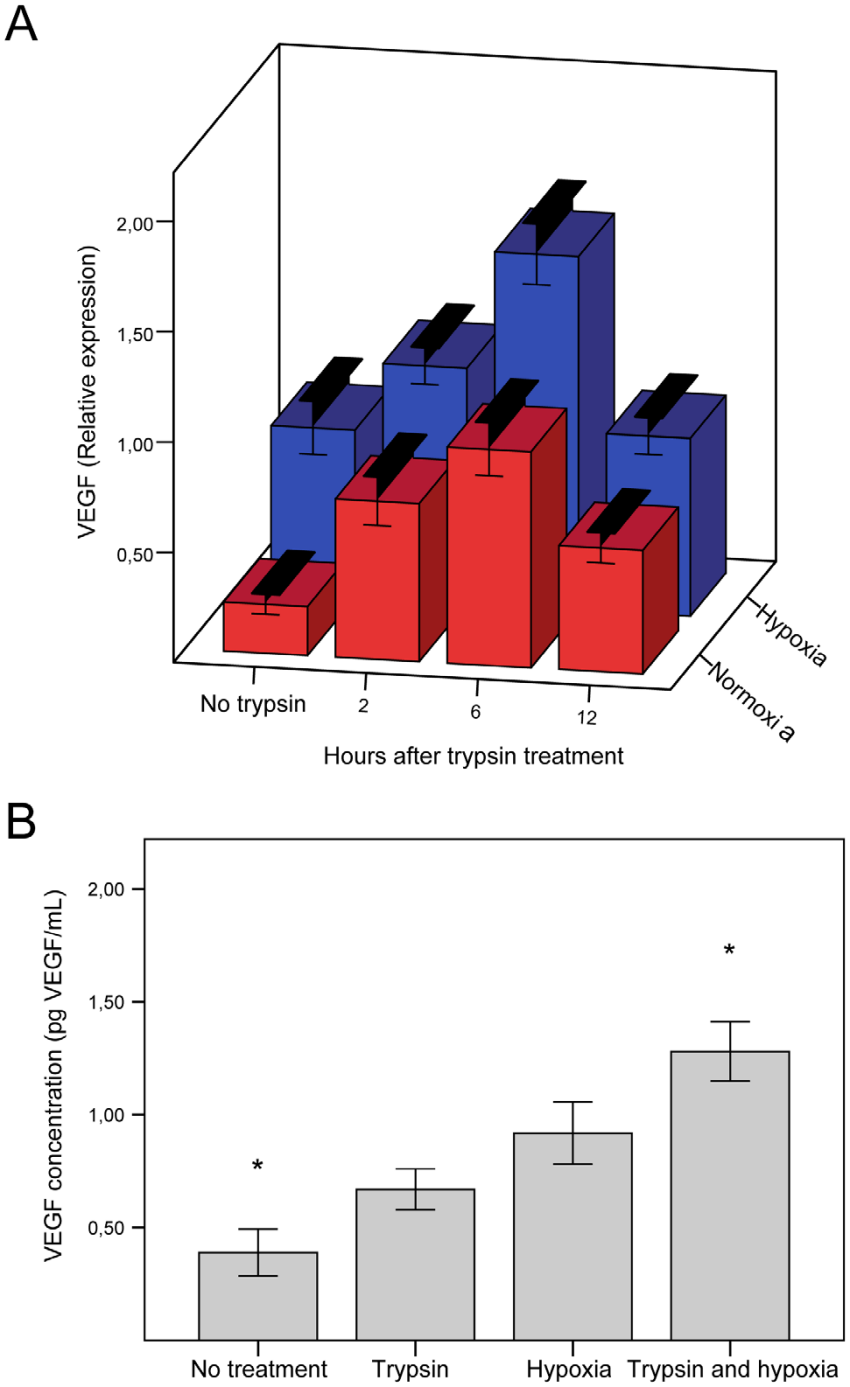
Effect of trypsin and culture at 1% oxygen on VEGF gene expression and secretion by hASCs. (**A**) The effect of trypsin exposure followed by various lengths of incubation alone or in combination with hypoxic exposure on *VEGF* gene expression was determined by real-time RT-PCR (n = 6–20). The transcriptional response was normalized to the geometric mean of reference genes PPIA and YWHAZ. Values are represented as the mean and SEM. For all time points the VEGF expression was significantly higher after trypsin treatment than in cells not treated with trypsin and also for all time points, the VEGF expression in hypoxia was significantly higher than the corresponding normoxic value (p<0.05) (**B**) Comparative analysis of the effect of trypsin or hypoxic exposure alone and in combination on VEGF secretion in the culture media was measured by ELISA (n = 12). Values are represented as the mean and SEM. Asterisks denote statistical difference between this and all other groups (p<0.05). Abbreviations: VEGF, vascular endothelial growth factor.

### Blocking of PAR2

Cells were seeded in 12-well plates (Costar) at a density of 8×10^3^ cells per cm^2^ and after three days the blocking of PAR2 was assayed using PAR2 (SAM11) (Santa Cruz Biotechnology) antibody at 25 µg/ml both before and during trypsin exposure for a total of 15 min. Cultures exposed to trypsin were used as positive controls and cultures exposed to PBS were used to normalise to the steady-state VEGF gene expression. After replenishing with complete growth medium, the cultures were allowed to recover for additional 12 hours, and finally processed for total RNA to enable real-time RT-PCR analysis.

### Inhibition of Kinase Activity

The cultures were initiated at a density of 8×10^3^ cells per cm^2^ and allowed to progress for three days, when the trypsin exposure was carried out. The selected kinase inhibitors, including 100 nM Wortmannin and 50 µM LY294002 (both phosphoinositide 3-kinase (PI3K)), 40 µM U0126 (mitogen activated protein kinase MEK), and 10 µM Y27632 (Rho-associated protein kinase (ROCK)), were allowed to take effect from one to two hours prior to enzymatic exposure. After supplementing with complete medium, the cultures were processed for protein analysis by immunoblotting and total RNA within 15 min and 12 hours, respectively.

**Figure 5:**
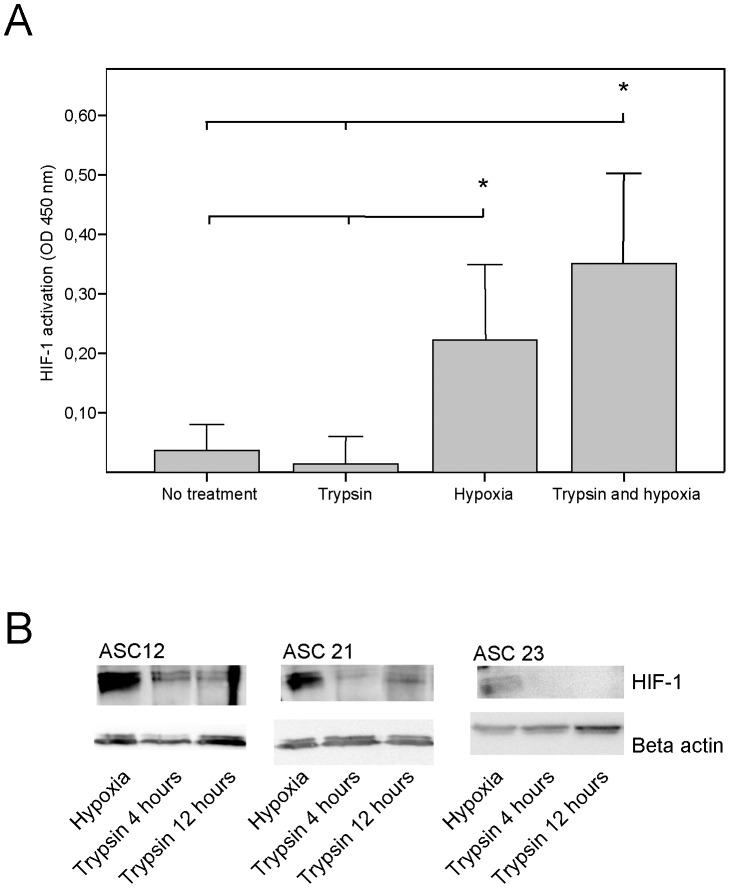
Stabilisation of HIF-1 in hASCs after trypsin and 1% oxygen exposure alone or in combination. (**A**) HIF-1 activation/stabilization during 6 hours culture after trypsin exposure in combination with 24 hours in hypoxic/normoxic conditions was analysed by ELISA. All cells were harvested *in situ*. Values are represented as the mean and SEM (n = 12). Asterisks denote statistical difference between this and all other groups (p<0.05). (**B**) Analysis of HIF-1α induction at 4 and 12 hours following 5 min trypsin exposure was done by immunoblotting. All cells were harvested *in situ*. HIF-1α positive controls are ASCs subjected to 48 hours of 1% oxygen.

### Immunoblotting

The cells were lysed in 50 mM Tris, 150 mM NaCl, 2 mM EDTA, 0.5% NP40, 0.5 mM phenylmethylsulfonyl fluoride, 50 mM NaF, 0.04% β-mercaptoethanol, 1 mM Na_3_VO_4_ and 1 Complete Mini protease inhibitor cocktail tablet (Roche Diagnostics, Hvidovre, Denmark) per 10 ml total lysis solution. Protein concentration was measured in the lysates using the BCA Protein Assay Kit (Thermo Fisher Scientific, Rockford, IL). Samples amounting to 40 to 60 µg of total protein were heat denatured, separated by SDS-PAGE using a 10% gel, and transferred to nitrocellulose or PVDF membranes using the iBlot transfer equipment (Invitrogen). Membranes were incubated at 4°C overnight with primary antibodies including rabbit polyclonal to HIF-1α (Abcam) diluted 1∶500, mouse monoclonal to β-actin (Sigma-Aldrich, Brøndby, Denmark) diluted 1∶10.000, rabbit monoclonals to Erk1/2, phospho-Erk1/2, Akt, and phospho-Akt (all from Cell Signaling Technology, Danvers, MA) diluted 1∶500, and finally mouse monoclonal to PAR2 (SAM11; Santa Cruz Biotechnology) diluted 1∶100. PBS containing 5% skimmed milk and 1% Tween 20 (Sigma-Aldrich) was used for all dilutions. Membranes were then incubated at room temperature for 1 hour with HRP-conjugated secondary polyclonal antibodies rabbit anti-mouse (Dako, Glostrup, Denmark) or goat anti-rabbit (Santa Cruz Biotechnology) diluted 1∶10.000 and 1∶2.500, respectively. The target proteins were visualised using chromogenic deposition (tetramethylbenzidine; Sigma-Aldrich) or enhanced chemiluminescence (Amersham ECL Plus; GE Healthcare Europe, Brøndby, Denmark). Signal acquisition in the latter case was accomplished in a Kodak Image Station 4000 mm Pro (Carestream Health Denmark, Skovlunde, Denmark).

### Real-time RT-PCR

Total RNA was isolated using the NucleoSpin RNA XS kit (Macherey-Nagel, Düren, Germany) and the purity and concentration were determined spectrophotometrically (Nanodrop; Thermo Science, Wilmington, DE). Reverse transcription was performed with the iScript cDNA synthesis kit (Bio-Rad, Copenhagen, Denmark). All primers (Table 1) were designed using the open source software Primer3 and produced by DNA Technology (Aarhus, Denmark). The amplification reactions were performed on a My-Cycler real-time PCR system (Bio-Rad) in a final volume of 25 µl containing 5 pmol of each primer, 0.25 µl cDNA using the SYBR Green PCR supermix (Bio-Rad). The thermal cycling protocol involved initial denaturation at 95°C for 3 min and was followed by 40 cycles of denaturation at 95°C for 10 sec and primer annealing and elongation for 30 sec at predetermined optimal temperature. To test for the specificity of the product, a melt curve function of the IQ5 Optical System Software 2.1 (Bio-Rad) was invoked. A four-fold serially diluted standard curve derived from a pool of all the cDNA samples was used to calculate relative expression of each gene. For normalisation purposes, the geometric mean of two reference genes, cyclophilin A (PPIA) and tyrosine 3/tryptophan 5-monooxygenase activation protein (YWHAZ), was used [29].

### Quantitation of Secreted VEGF

For all treatments, after the medium was harvested the cells were lysed in 0.02% sodium dodecyl sulfate. The levels of VEGF were assessed using a commercially available ELISA VEGF kit (R&D Systems Europe, Abingdon, United Kingdom) and the cell numbers were determined using a PicoGreen dsDNA Quantitation Kit (Invitrogen). The fluorescence was measured using a Wallac 1420 Victor Multilabel Counter (PerkinElmer, Hvidovre, Denmark) with excitation and emission at 485 nm and 535 nm, respectively. The secretion of VEGF was normalised with respect to cell numbers.

### Quantitation of HIF-1

To investigate the influence of trypsin treatment on the activation of HIF-1, the ASCs were seeded in duplicates in T75 Cellstar® Tissue Culture Flasks (Greiner Bio-One, Cat. no. 658175) and incubated under standard culturing conditions for three days. Half of the cells were transferred to an incubator in the BioSpherix glove box to receive hypoxic treatment. 6 h before the hypoxic treatment was completed a group of cells from each incubator system were treated with trypsin. After completion of the hypoxic treatment, all cells were lysed and nuclear proteins isolated using a Nuclear Extract Kit (Active Motif, Cat. no. 40010) and the concentration of nuclear extract determined using a Pierce BCA Protein Assay Kit (Thermo Scientific, Cat. no. 23227) according to manufacturer’s instructions. Nuclear extract was kept at –80°C.

The HIF-1 activation was measured in technical replicates using a TransAM HIF-1 kit (Active Motif, Cat. no. 47096). For each sample 20 µg was used per well. The absorbance was measured using a Wallac 1420 Victor Multilabel Counter (PerkinElmer, Hvidovre, Denmark) at a wavelength of 450 nm.

### Statistics

When comparing more than two groups a one-way analysis of variance (ANOVA) with Bonferroni’s post hoc test was used and when comparing two samples, a Student’s t-test was used (SigmaPlot 11.0; Systat Software, Erkrath, Germany). When necessary, data were logarithmic transformed to display normal distribution. Data are presented as mean ± standard error of the mean (SEM). Statistical significance was assigned to p values <0.05. The experiments were done using all three cell lines and when quantitative parameters were assessed the experiments were repeated at least twice and replicates were involved.

## Results

### Characterization of hASCs

FACS analyses of all three cell lines demonstrated that the hASC cell lines were positive for the surface markers CD29, CD44, CD73, CD90, CD105, and HLA-ABC and negative for CD24, CD34, CD45, CD117, CD133, CD146, CD200, CD271, ABCG2, HLA-DR, and Stro-1 (Fig. 1A and 1B). Moreover, a narrow distribution of the marker values indicated a phenotypical relatedness of the cell lines. In addition, the particular cell lines displayed plastic adherence and have previously been demonstrated to have a multilineage potential. [28], [30] These characteristics apply with the general accepted definitions of ASCs [31], [32].

### Role of PAR2 in Trypsin-mediated Upregulation of VEGF

Initially, we investigated whether PAR2 is expressed by hASCs. Immunoblot analysis revealed the presence of the PAR2 protein (Fig. 2A) and, furthermore, immunofluorescence indicated that PAR2 was localised at the plasma membrane (Fig. 2B). After having confirmed the expression of PAR2, we aimed to link its activation to upregulation of VEGF. To that end, we carried out experiments where PAR2 was blocked by applying a specific antibody. Blocking of PAR2 receptors resulted in a significant suppression of trypsin-induced *VEGF* transcriptional upregulation (Fig. 2C).

### Identification of Molecular Mechanisms Underlying PAR2 Mediated VEGF Induction

In order to identify molecular mechanisms downstream of PAR2, we explored involvement of the kinase cascades PI3K, MAPK, and ROCK that are known to be more or less involved in PAR2-specific signal transduction in other cell lines (Fig. 3A). The blocking experiments revealed that the transcriptional activation of *VEGF* in hASCs in response to trypsin could be attenuated by blocking the PI3K and MAPK pathways while blocking the ROCK pathway had only minor effect (Fig. 3B). This finding was further confirmed by immunoblotting experiments, demonstrating that trypsin-dependent phosphorylation of Akt and Erk1/2 took place, and that this effect was blocked by specific inhibitors of PI3K and MEK (Fig. 3C).

### Induction of VEGF in Response to Trypsin and Hypoxia

To determine the optimal conditions of trypsin treatment, first several concentrations of trypsin were tested. When *VEGF* expression levels were normalized to a PBS exposed control, 9 nM, 90 nM, and 9,000 nM trypsin upregulated *VEGF* 1.12, 1.21, and 1.52 times respectively with only the 9,000 nM trypsin resulting in a significant upregulation (p<0.05). (Data not shown).

To determine when the expression of VEGF peaked after trypsin treatment, cells were incubated for 2, 6, and 12 hours after trypsin treatment, after which VEGF mRNA levels were determined. In addition, to assess the combined effect of trypsin and hypoxia this series of experiments was performed both at ambient oxygen and under hypoxic conditions. The data demonstrate the expression of VEGF peaks 6 hours after trypsin treatment (Fig. 4A, red bars) and that for all time points hypoxia has an additional effect (Fig. 4A, blue bars).

The additive effect of trypsin and hypoxia was also evident when looking at the amount of secreted VEGF into the media. Both trypsin and hypoxic treatments alone led to significantly higher levels of secreted VEGF than control conditions, and the combination of the two resulted in the highest level of VEGF secretion (Fig. 4B).

### Effect of Trypsin and Hypoxia on HIF-1 Induction

HIF-1α is responsible for *VEGF* upregulation in hypoxia, therefore we investigated the possibility that trypsin-induced upregulation of *VEGF* was effectuated through stabilisation of HIF-1α. Cells were either subjected to trypsin, hypoxia or a combination, after which HIF-1 activation/stabilization was determined by ELISA (Fig. 5A). Both hypoxia and trypsin + hypoxia led to significantly increased levels of HIF-1. However, treatment with trypsin failed to increase HIF-1 compared to untreated cells. The cells in this first experiment were cultured for 6 hours after trypsin exposure. Thus, to exclude the possibility that HIF-1α could have been stabilised in short term in the period preceding or following this time point. cells were exposed to trypsin and cultured for 4 or 12 hours prior to analysis of HIF-1 levels by immunoblotting (Fig. 5B). Cells cultured for 24 hours in hypoxic conditions were used as a positive control. As shown in the panels in figure 5B, for all cell lines HIF-1α was stabilised in 1% oxygen culture and HIF-1 levels in trypsin-treated cells both 4 and 12 hours after trypsin treatment remained negligible. Cells receiving no treatment had HIF-1 levels comparable to the cells treated with trypsin (data not shown).

## Discussion

In this study, we have found that PAR2 is expressed on the surface of hASCs, that trypsin induces the expression of VEGF via PAR2 activation and subsequent activation of PI3K and MEK signalling pathways, and that this mechanism is independent of HIF-1.

We are the first to demonstrate the presence of PAR2 on hASCs, and to document that in hASCs the PAR2 activation leads to downstream activation of PI3K and MAPK pathways, and that the Rho/ROCK pathway is minimally involved. This finding underscores the interesting observation that PAR2 activation has very distinct down-stream effects depending on the cell type, as in some cell types only the MAPK or the Rho/ROCK pathways are activated [9], [18], [19].

Furthermore, the additive effect of hypoxia and trypsin treatment on VEGF induction has not been described previously either. Several other groups have shown that activation of PI3K and MAPK pathways may lead to either stabilization or increased expression of HIF-1 [33], [34]. Therefore, we speculated that the trypsin-induced expression of VEGF may be mediated via HIF-1 involvement. However, as we demonstrate here for the first time in ASCs, trypsin-treatment alone failed to stabilize HIF-1a. Therefore, it can be concluded that the hypoxia-induced and trypsin-induced VEGF transcription occurs through separate pathways.

The effect of hASCs transplantation in the treatment of ischemic disease is believed to be mainly paracrine, with VEGF playing a key role [1], [2], [5]. Therefore, it is an important discovery that trypsin through PAR2 induces a level of VEGF upregulation in hASCs comparable to that seen in hASCs cultured at 1% oxygen. In addition, the present investigation, demonstrating synergistic effects of hypoxia and trypsin on VEGF induction, may lay the basis for a novel paradigm for preconditioning of the hASCs by a combination of hypoxic culture and exposure to trypsin prior to their clinical application. We expect that an optimised implementation of exposure to a combination of hypoxia and trypsin will form the basis for a highly efficient preconditioning which will impact cellular therapies for ischemic disorders. The clinical implications of these findings are significant as ischemic disease is a major burden in the health care system and the deleterious effects of ischemia could be ameliorated by inducing angiogenesis in the ischemic tissue using ASCs with potentiated VEGF secretion.

## References

[1] Bai X, Alt E (2010) Myocardial regeneration potential of adipose tissue-derived stem cells. Biochemical and biophysical research communications 401: 321–326. 20833143 10.1016/j.bbrc.2010.09.012

[2] Miranville A, Heeschen C, Sengenès C, Curat CA, Busse R, et al (2004) Improvement of postnatal neovascularization by human adipose tissue-derived stem cells. Circulation 110: 349–355. 15238461 10.1161/01.CIR.0000135466.16823.D0

[3] Zachar V, Duroux M, Emmersen J, Rasmussen JG, Pennisi CP, et al (2011) Hypoxia and adipose-derived stem cell-based tissue regeneration and engineering. Expert opinion on biological therapy 11: 775–786. 21413910 10.1517/14712598.2011.570258

[4] Rasmussen JG, Frøbert O, Pilgaard L, Kastrup J, Simonsen U, et al (2011) Prolonged hypoxic culture and trypsinization increase the pro-angiogenic potential of human adipose tissue-derived stem cells. Cytotherapy 13: 318–328. 20795759 10.3109/14653249.2010.506505

[5] Sadat S, Gehmert S, Song Y-H, Yen Y, Bai X, et al (2007) The cardioprotective effect of mesenchymal stem cells is mediated by IGF-I and VEGF. Biochemical and biophysical research communications 363: 674–679. 17904522 10.1016/j.bbrc.2007.09.058

[6] Rehman J, Traktuev D, Li J, Merfeld-Clauss S, Temm-Grove CJ, et al (2004) Secretion of angiogenic and antiapoptotic factors by human adipose stromal cells. Circulation 109: 1292–1298. 14993122 10.1161/01.CIR.0000121425.42966.F1

[7] Herrmann JL, Wang Y, Abarbanell AM, Weil BR, Tan J, et al (2010) Preconditioning mesenchymal stem cells with transforming growth factor-alpha improves mesenchymal stem cell-mediated cardioprotection. Shock (Augusta, Ga) 33: 24–30. 10.1097/SHK.0b013e3181b7d137 19996917

[8] Wang X, Zhao T, Huang W, Wang T, Qian J, et al (2009) Hsp20-engineered mesenchymal stem cells are resistant to oxidative stress via enhanced activation of Akt and increased secretion of growth factors. Stem cells (Dayton, Ohio) 27: 3021–3031. 10.1002/stem.230 PMC2806498 19816949

[9] Liu Y, Mueller BM (2006) Protease-activated receptor-2 regulates vascular endothelial growth factor expression in MDA-MB-231 cells via MAPK pathways. Biochemical and biophysical research communications 344: 1263–1270. 16650817 10.1016/j.bbrc.2006.04.005

[10] Zhu T, Sennlaub F, Beauchamp MH, Fan L, Joyal JS, et al (2006) Proangiogenic effects of protease-activated receptor 2 are tumor necrosis factor-alpha and consecutively Tie2 dependent. Arteriosclerosis, thrombosis, and vascular biology 26: 744–750. 10.1161/01.ATV.0000205591.88522.d4 16439712

[11] Forsythe JA, Jiang BH, Iyer NV, Agani F, Leung SW, et al (1996) Activation of vascular endothelial growth factor gene transcription by hypoxia-inducible factor 1. Molecular and cellular biology 16: 4604–4613. 8756616 10.1128/mcb.16.9.4604 PMC231459

[12] Nystedt S, Emilsson K, Wahlestedt C, Sundelin J (1994) Molecular cloning of a potential proteinase activated receptor. Proceedings of the National Academy of Sciences of the United States of America 91: 9208–9212. 7937743 10.1073/pnas.91.20.9208 PMC44781

[13] Déry O, Thoma MS, Wong H, Grady EF, Bunnett NW (1999) Trafficking of proteinase-activated receptor-2 and beta-arrestin-1 tagged with green fluorescent protein. beta-Arrestin-dependent endocytosis of a proteinase receptor. The Journal of biological chemistry 274: 18524–18535. 10373461 10.1074/jbc.274.26.18524

[14] Vu TK, Hung DT, Wheaton VI, Coughlin SR (1991) Molecular cloning of a functional thrombin receptor reveals a novel proteolytic mechanism of receptor activation. Cell 64: 1057–1068. 1672265 10.1016/0092-8674(91)90261-v

[15] Greenberg DL, Mize GJ, Takayama TK (2003) Protease-activated receptor mediated RhoA signaling and cytoskeletal reorganization in LNCaP cells. Biochemistry 42: 702–709. 12534282 10.1021/bi027100x

[16] Kawabata A, Saifeddine M, Al-Ani B, Leblond L, Hollenberg MD (1999) Evaluation of proteinase-activated receptor-1 (PAR1) agonists and antagonists using a cultured cell receptor desensitization assay: activation of PAR2 by PAR1-targeted ligands. The Journal of pharmacology and experimental therapeutics 288: 358–370. 9862790

[17] Tanaka Y, Sekiguchi F, Hong H, Kawabata A (2008) PAR2 triggers IL-8 release via MEK/ERK and PI3-kinase/Akt pathways in GI epithelial cells. Biochemical and biophysical research communications 377: 622–626. 18854173 10.1016/j.bbrc.2008.10.018

[18] Yagi Y, Otani H, Ando S, Oshiro A, Kawai K, et al (2006) Involvement of Rho signaling in PAR2-mediated regulation of neutrophil adhesion to lung epithelial cells. European journal of pharmacology 536: 19–27. 16564523 10.1016/j.ejphar.2006.02.024

[19] Dutra-Oliveira A, Monteiro RQ, Mariano-Oliveira A (2012) Protease-activated receptor-2 (PAR2) mediates VEGF production through the ERK1/2 pathway in human glioblastoma cell lines. Biochemical and biophysical research communications 421: 221–227. 22497886 10.1016/j.bbrc.2012.03.140

[20] Camerer E, Barker A, Duong DN, Ganesan R, Kataoka H, et al (2010) Local protease signaling contributes to neural tube closure in the mouse embryo. Developmental cell 18: 25–38. 20152175 10.1016/j.devcel.2009.11.014 PMC2822780

[21] Brahimi-Horn MC, Pouysségur J (2009) HIF at a glance. Journal of cell science 122: 1055–1057. 19339544 10.1242/jcs.035022

[22] Leung K-W, Ng H-M, Tang MKS, Wong CCK, Wong RNS, et al (2011) Ginsenoside-Rg1 mediates a hypoxia-independent upregulation of hypoxia-inducible factor-1α to promote angiogenesis. Angiogenesis 14: 515–522. 21964931 10.1007/s10456-011-9235-z PMC3214261

[23] Jing Y, Liu L-Z, Jiang Y, Zhu Y, Guo NL, et al (2012) Cadmium increases HIF-1 and VEGF expression through ROS, ERK, and AKT signaling pathways and induces malignant transformation of human bronchial epithelial cells. Toxicological sciences: an official journal of the Society of Toxicology 125: 10–19. 21984483 10.1093/toxsci/kfr256 PMC3243743

[24] Uusitalo-Jarvinen H, Kurokawa T, Mueller BM, Andrade-Gordon P, Friedlander M, et al (2007) Role of protease activated receptor 1 and 2 signaling in hypoxia-induced angiogenesis. Arteriosclerosis, thrombosis, and vascular biology 27: 1456–1462. 10.1161/ATVBAHA.107.142539 17363687

[25] Svensson KJ, Kucharzewska P, Christianson HC, Sköld S, Löfstedt T, et al (2011) Hypoxia triggers a proangiogenic pathway involving cancer cell microvesicles and PAR-2-mediated heparin-binding EGF signaling in endothelial cells. Proceedings of the National Academy of Sciences of the United States of America 108: 13147–13152. 21788507 10.1073/pnas.1104261108 PMC3156184

[26] Pilgaard L, Lund P, Rasmussen JG, Fink T, Zachar V (2008) Comparative analysis of highly defined proteases for the isolation of adipose tissue-derived stem cells. Regenerative medicine 3: 705–715. 18729795 10.2217/17460751.3.5.705

[27] Fink T, Rasmussen JG, Lund P, Pilgaard L, Soballe K, et al (2011) Isolation and expansion of adipose-derived stem cells for tissue engineering. Frontiers in bioscience (Elite edition) 3: 256–263. 21196306 10.2741/e241

[28] Pilgaard L, Lund P, Duroux M, Fink T, Ulrich-Vinther M, et al (2009) Effect of oxygen concentration, culture format and donor variability on in vitro chondrogenesis of human adipose tissue-derived stem cells. Regenerative medicine 4: 539–548. 19580403 10.2217/rme.09.28

[29] Fink T, Lund P, Pilgaard L, Rasmussen JG, Duroux M, et al (2008) Instability of standard PCR reference genes in adipose-derived stem cells during propagation, differentiation and hypoxic exposure. BMC molecular biology 9: 98. 18976469 10.1186/1471-2199-9-98 PMC2585587

[30] Fink T, Zachar V (2011) Adipogenic differentiation of human mesenchymal stem cells. Methods in molecular biology (Clifton, NJ) 698: 243–251. 10.1007/978-1-60761-999-4_19 21431524

[31] Dominici M, Le Blanc K, Mueller I, Slaper-Cortenbach I, Marini F, et al (2006) Minimal criteria for defining multipotent mesenchymal stromal cells. The International Society for Cellular Therapy position statement. Cytotherapy 8: 315–317. 16923606 10.1080/14653240600855905

[32] Zuk PA, Zhu M, Ashjian P, De Ugarte DA, Huang JI, et al (2002) Human adipose tissue is a source of multipotent stem cells. Molecular biology of the cell 13: 4279–4295. 12475952 10.1091/mbc.E02-02-0105 PMC138633

[33] Zhong H, Chiles K, Feldser D, Laughner E, Hanrahan C, et al (2000) Modulation of hypoxia-inducible factor 1alpha expression by the epidermal growth factor/phosphatidylinositol 3-kinase/PTEN/AKT/FRAP pathway in human prostate cancer cells: implications for tumor angiogenesis and therapeutics. Cancer research 60: 1541–1545. 10749120

[34] Fukuda R, Hirota K, Fan F, Jung YD, Ellis LM, et al (2002) Insulin-like growth factor 1 induces hypoxia-inducible factor 1-mediated vascular endothelial growth factor expression, which is dependent on MAP kinase and phosphatidylinositol 3-kinase signaling in colon cancer cells. The Journal of biological chemistry 277: 38205–38211. 12149254 10.1074/jbc.M203781200

